# Eucharitidae (Hymenoptera, Chalcidoidea), a family new to the fauna of Saudi Arabia, with the description of the previously unknown male of Eucharis (Psilogastrellus) affinis Bouček

**DOI:** 10.3897/zookeys.462.8437

**Published:** 2014-12-10

**Authors:** Neveen S. Gadallah, Yusuf A. Edmardash, Hathal M. Al Dhafer, Magdi S. El-Hawagry

**Affiliations:** 1 Entomology Department, Faculty of Science, Cairo University, Egypt; 2 Museum of Arthropods (KSMA), Plant Protection Department, College of Food and Agriculture Sciences, King Saud University, Riyadh, Saudi Arabia

**Keywords:** Eucharitidae, *Eucharis*, *Hydrorhoa*, new records, Baha City, Asir province, Saudi Arabia

## Abstract

The family Eucharitidae (Hymenoptera: Chalcidoidea) is recorded for the first time for the fauna of Saudi Arabia based on *Hydrorhoa caffra* (Westwood) and Eucharis (Psilogastrellus) affinis Bouček. The record of *Hydrorhoa caffra* suggests that Al-Baha and Asir provinces should be considered as part of the Afrotropical rather than the Palaearctic region. The previously unknown male of *Eucharis affinis* Bouček is described and figured. Macrophotographs of the species are provided.

## Introduction

The Eucharitidae (Hymenoptera: Chalcidoidea) are a monophyletic family of solitary parasitoids of ant pupae (Clausen 1940, Heraty 2002). They are the largest and most diverse group of hymenopteran parasitoids of eusocial insects. Members are distributed in almost every zoogeographic region of the world but are most abundant in the tropics (Heraty 2002). The family includes 54 genera and about 420 species worldwide (Heraty 2002, Torréns 2013, Torréns and Heraty 2013) classified in the three subfamilies Gollumiellinae, Oraseminae and Eucharitinae (Munro et al. 2011, Heraty et al. 2013, Murray et al. 2013).

The family is poorly represented in the Arabian Peninsula, with only two species of *Stilbula* Spinola (Narendran and Kumar 2004) and a single species of *Orasema* Cameron (Heraty 1994) reported from Yemen.

The Eucharitidae are here reported from the Arabian Peninsula, from Saudi Arabia (Al-Mekhwah, Baha Province; Raydah, Abha, Asir Province) based on Eucharis (Psilogastrellus) affinis Bouček, for which we describe and figure the male for the first time, and *Hydrorhoa caffra* (Westwood).

Al-Baha and Asir provinces are situated in the south-western part of the kingdom of Saudi Arabia and are characterized by a natural tree cover and agricultural figaus. Both are similarly divided into two main sectors. A lowland in the west forms a part of the coastal plain that extends from north to south, which is known as “Tihama”. There is also a mountainous area in the east with an elevation of about 1500-3000 m above sea level, known as “Al-Sarat or Al-Sarah” which forms part of the Al-Sarawat mountain range (Alahmed et al. 2010, Ibrahim and Abdoon 2005, El-Hawagry et al. 2013).

Sclater (1858) and Wallace (1876) were the first to propose many of the classical zoogeographic regions, and they placed the northern border of the Afrotropical region along the Tropic of Cancer. Consequently, Al-Baha and Asir Provinces were included in the Afrotropical region (Hölzel 1998). However, according to Uvarov (1938), Greathead (1980), and Larsen (1984) this area should be united with the central Arabian deserts which are either considered as a part of the Palaearctic or, by some authors, as an autonomous Eremic zone (also called the Saharo-Sindian faunal region).

## Material and methods

This study is based on specimens collected by sweep net in Saudi Arabia, from Al-Mekhwah (Baha City) and Raydah, Abha (Asir province). The male of *Hydrorhoa caffra* is deposited in Museum of Arthropods, Plant Protection Department, Faculty of Food and Agriculture Sciences, King Saud University, KSA (KSMA), while that of Eucharis (Psilogastrellus) affinis is deposited in the Efflatoun Bey collection, Entomology Department, Faculty of Science, Cairo University (CUE). Morphological terms are based on Gibson (1997) and Heraty (1992, 2002, 2004). Terminology for body sculpture follows Harris (1979). Photos were taken with a Canon camera (G12) attached to an Optech trinocular zoom streomicroscope (LFZT). Measurements of the different parts were made with the help of an ocular micrometer.

**Abbreviations used in the text are:**

F = flagellomere; GS9 = last male sternite; OOL = ocellocular line; POL = posterior ocellar line; T = metasomal tergite.

**Insect depositories mentioned in the text:**

CUE = Efflatoun Bey collection (Entomology Department, Faculty of Science, Cairo University, Egypt); KSMA = Museum of Arthropods, Plant Protection Department, Faculty of Food and Agriculture Sciences, King Saud University, Saudi Arabia; NMPC = National Museum, Prague, Czech Republic; SAMC = South African Museum, Cape Town; UMOX = Hope Entomological Collections, Oxford University Museum of Natural History, Oxford, England.

### Hydrorhoa caffra

Taxon classificationAnimaliaHymenopteraEucharitidae

(Westwood, 1874)

Figs 1–3

Schizaspidia caffra Westwood, 1874: 152. Type data: Africa, Caffraria [South Africa]. Holotype ♂, by monotypy. Type depository: UMOX.
Stibulaspis fortistriata Cameron, 1907: 221. Type data: South Africa, KwaZulu-Natal, Estcourt. Lectotype ♀, designation by Heraty 2002 for nomenclatural stability. Type depository: SAMC.
Stibulaspis atropurpurea Cameron, 1907: 221–222. Type data: South Africa: KwaZulu-Natal, Durban. Lectotype ♂, designated by Heraty 2002 for nomenclatural stability. Type depository: SAMC.
Hydrorhoa caffra ; Heraty 2002: 162.

#### Material examined.

Raydah (Abha, Asir province), 18° 11.88'N; 42° 24.44'E, 2387 m, 7.vi.2014, leg. El-Hawagry [1♂, KSMA].

#### Diagnosis.

**Figures 1–3.**
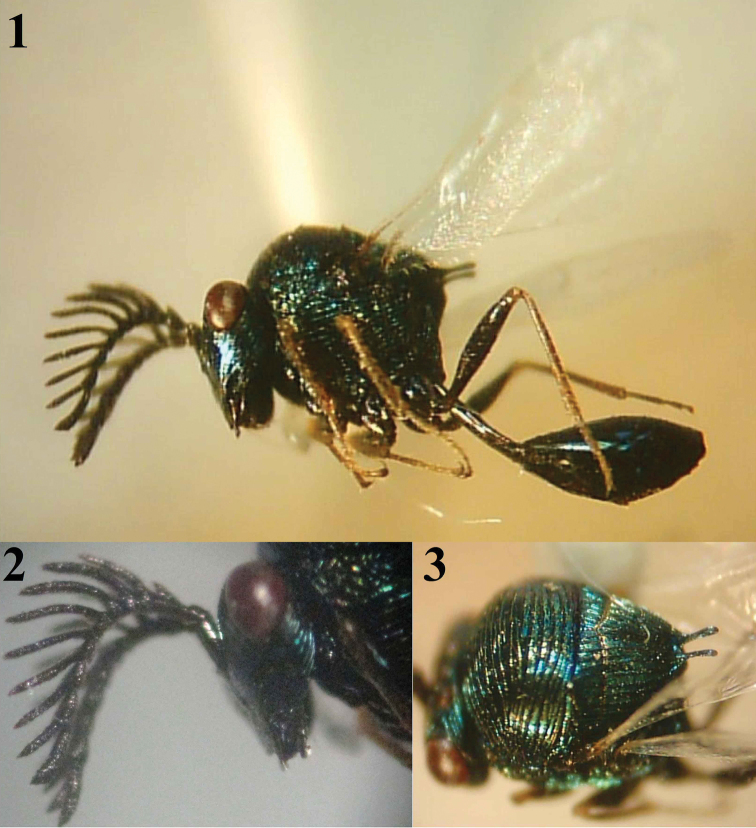
*Hydrorhoa caffra* (Westwood) (♂): **1** General habitus **2** Lateral view of head **3** Dorso-lateral aspect of mesosoma.

Both sexes of this species are characterized within the genus by the form of the scutellar spines (Heraty 2002). The male is characterized by the following: metallic bluish-green color (Fig. 1); flagellomeres with long pectination; malar space long (about 2× longitudinal diameter of eye), transversely striated (Fig. 2); mesoscutum with coarse transverse striations and scutellum with coarse longitudinal striations (Fig. 3); posterior margin of scutellum with two long, slender processes, the distance between them less than half length of either (Fig. 3).

#### Distribution.

Afrotropical: Kenya, Zimbabwe (Heraty 2002), South Africa (Westwood 1874, Cameron 1907, Heraty 2002), new to the Arabian Peninsula.

#### Remarks.

*Hydrorhoa caffra* has Afrotropical affinities, previously being recorded only from Kenya, Zimbabwe and South Africa. The present record in Saudi Arabia is significant because it supports studies which consider that parts of the Arabian Peninsula, including Al-Baha and Asir Provinces, should be included in the Afrotropical rather than in the Palaearctic region or the Eremic zone, and that the northern limit of the Afrotropical region should be placed in the Taif area, about 200 km north of Al-Baha (El-Hawagry et al. 2013, Hölzel 1998, Sclater 1858, Wallace 1876).

### Eucharis (Psilogastrellus) affinis

Taxon classificationAnimaliaHymenopteraEucharitidae

Bouček, 1956 (male)

Figs 4–9

Eucharis (Pachyeucharis) affinis Bouček, 1956: 255–256. Type data: Israel: Bat Yam. Holotype ♀, by original designation. Type depository: NMPC. Description of female, with illustrations.
Psilogastrellus affinis ; Bouček 1977: 124. Change of combination (by inference).
Eucharis (Psilogastrellus) affinis ; Heraty 2002: 144.

#### Material examined.

Saudi Arabia (Al-Mekhwah, Baha Province), 19°48.81'N; 41°26.45'E, 455m, 6.vii.2012, leg. El-Hawagry [1♂, CUE].

#### Description.

**Figures 4–9.**
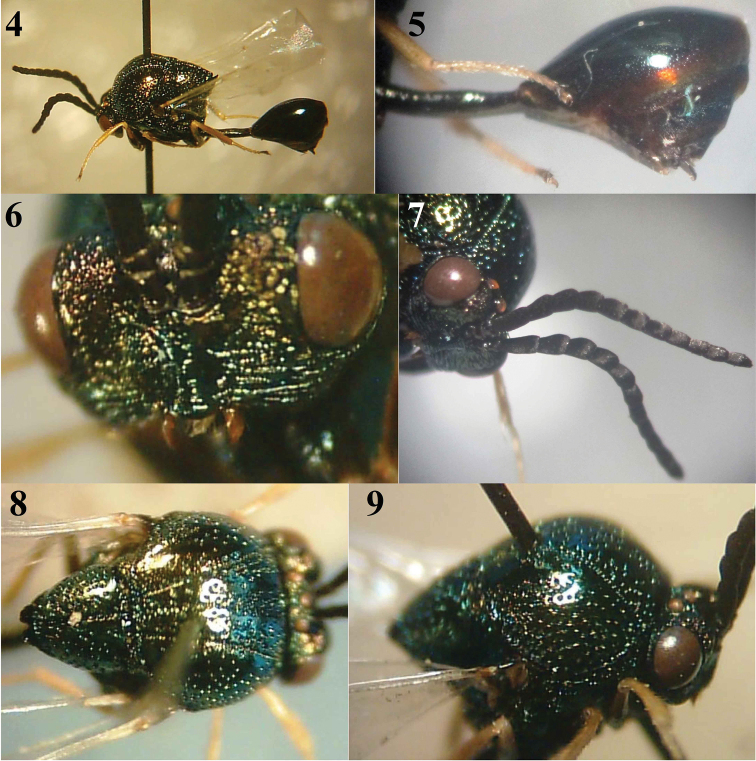
Eucharis (Psilogastrellus) affinis Bouček (♂): **4** General habitus **5** Lateral aspect of metasoma **6** Frontal view of head **7** Antenna **8** Dorsal view of mesososoma **9** Lateral aspect of mesosoma.

**Male:** Body length: 6.1 mm. *Coloration* (Fig. 4). Head metallic dark green (except for upper face between eyes to lateral ocelli, antennal scape and pedicel which are metallic purple, flagellum dark brown to black, mandible reddish to orange, with black tip); mesosoma dark bluish green with reddish reflections especially lateral lobes; legs yellow except coxae which are metallic dark green; trochanters, basal two thirds of front and hind femora, middle femur (except distally), last tarsomeres and claws dark brown to black; metasoma brilliant metallic green, with a broad fulvous apical band on T1 as well as narrower bands on posterior margins of remaining tergites (Fig. 5); metasomal sternites mostly fulvous. Wings hyaline, with inconspicuous pale brown veins.

*Head*: In dorsal view semiglobular to transverse, 1.6× as broad as high and narrower than mesosoma (8:10). POL 1.7× OOL. Lower face, malar space and clypeus coarsely transversely striate. Clypeus not separated from supraclypeal area (Fig. 6), clypeal margin rounded. Eyes separated by 2.2× their height. Malar space 0.4× height of eye. Ocelli on uppermost part of head, equal in size, arranged in a broadly obtuse ocellar triangle forming an almost straight transverse line, interocular area with very thin transverse striations. Antenna (Fig. 7) 13-segmented, flagellomeres closely appressed; scape length 0.8× distance between torulus and upper margin of median ocellus; all flagellomeres longer than broad and cylindrical, F1 1.5× as long as F2 and F3 equal, F6-8 slightly longer than broad, last flagellomere rhomboidal, acuminate distally. Mandible relatively short, sickle-shaped ventrally and lacking subapical teeth.

*Mesosoma* (Figs 8, 9): Glabrous, with irregularly dispersed fine punctures, denser anteriorly and laterally. Mesoscutum with notauli well developed as foveolate lines, extending along its whole length, becoming thin anteriorly and more distinct, thicker and converging posteriorly; with a longitudinal foveolate sulcus between notauli, this sulcus indistinct on anterior half of mesoscutum, deeper and more distinct on posterior half, extending through scutellum along longitudinal mid line where it is deeper than on mesoscutum. Scutellar disc relatively large, hardly longer than broad, with closer and deeper puncturations dorsally, and with two close sub-triangular posterior processes, the distance between them less than length of either. Propodeum conical, medially depressed, with dense thick transverse striations laterally. Prepectus densely finely punctate. Mesopleuron shiny but superficially micropunctate, and smooth and shiny ventrally; metapleuron with dense thick transverse striations. Front coxa coarsely sculptured, mid and hind coxae smooth and shiny. Wings with inconspicuous veins.

*Metasoma* (Fig. 5). Petiole relatively long, 2.1× as long as hind coxa and 5.9× as long as broad, straight in profile, with a fine irregular sculpture, dorsally with a narrow median sulcus that becomes broader posteriorly. Metasomal T1 smooth, shagreened along posterior margin; T2–4 with dense fine, shallow punctures; epipygium very small, whitish; GS9 spoon-shaped, distinctly concave ventrally, pointed apically.

#### Distribution.

Israel (female, Bouček 1956), new for Arabian Peninsula.

#### Remarks.

**Figures 10–15.**
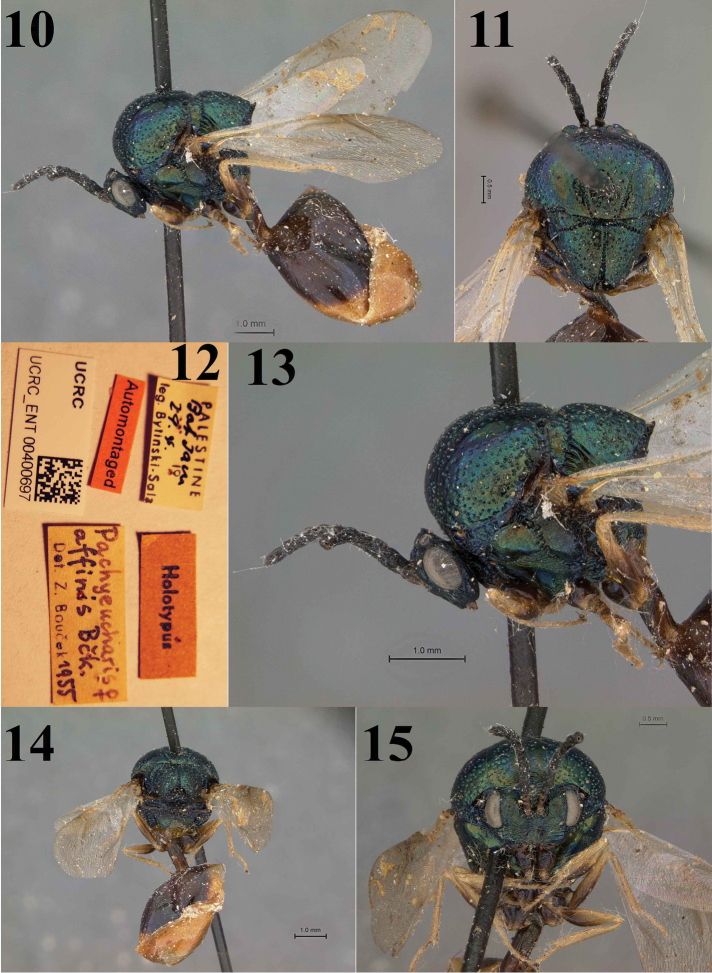
Eucharis (Psilogastrellus) affinis Bouček (♀ holotype): **10** Female holotype habitus **11** Dorsal view of mesosoma **12** Label (holotype ♀) **13** Lateral aspect of mesosoma **14** Posterior view of body **15** Frontal view of head.

The male resembles the female (Figs 10–15) described by Bouček (1956) except for the following: wings of male entirely hyaline without any infuscation (apical two thirds of wings infuscated in female); F6–8 slightly longer than broad (F7–12 subquadrate in female); metasoma mostly bright metallic green, with fulvous bands on posterior margin of tergites (metasoma mostly fulvous in female); unlike female, upper face metallic purplish; clypeus transversely striated (nearly smooth in female).

## Supplementary Material

XML Treatment for Hydrorhoa caffra

XML Treatment for Eucharis (Psilogastrellus) affinis
